# On exploiting Data Visualization and IoT for Increasing Sustainability and Safety in a Smart Campus

**DOI:** 10.1007/s11036-021-01742-4

**Published:** 2021-03-23

**Authors:** Chiara Ceccarini, Silvia Mirri, Paola Salomoni, Catia Prandi

**Affiliations:** Dipartimento di Informatica - Scienza e Ingegneria, Università di Bologna, Mura Anteo Zamboni 7, Bologna, 40126 Italy

**Keywords:** Smart campus, Data visualization, Sustainability, IoT

## Abstract

In a world that is getting increasingly digital and interconnected, and where more and more physical objects are integrated into the information network (Internet of Things, IoT), Data Visualization can facilitate the understanding of huge volumes of data. In this paper, we present the design and implementation of a testbed where IoT and Data Visualization have been exploited to increase the sustainability and safety of the Cesena (Smart) Campus. In particular, we detail the overall system architecture and the interactive dashboard that facilitates the management of the campus premises and the timetabling. Exploiting our system, we show how we can improve the campus sustainability (in terms of energy saving) and safety (considering the COVID-19 restrictions and regulations).

## Introduction

Data visualization can be defined as *“a way to represent information graphically, highlighting patterns and trends in data and helping the reader to achieve quick insights”* and as a tool to enable *“the exploration of data via the manipulation of chart images, with the color, brightness, size, shape, and motion of visual objects representing aspects of the dataset being analyzed”* [1]. In a world that is getting increasingly digital and interconnected, and where also the physical objects are integrated into the information network to collect data about the environment (Internet of Things, IoT), Data Visualization can facilitate the understanding of huge volumes of data. This is particularly true if related to smart environment, both outdoor (e.g., visualizing urban accessibility data [2–4]) and indoor (e.g., designing infographics to stimulate environmental awareness [5]). Focusing on the indoor, Data Visualization can be useful to display information tied to the IoT infrastructure and sensors deployed inside smart buildings, to make people aware of the specific conditions (e.g., air quality and energy consumption [6]) and dynamics (e.g., people flow, [7]) of the environment they are experiencing.

A particular case of a smart building is a smart campus [8, 9]. In this scenario, several studies investigated the use of data visualization, exploiting the campus as a user-centric testbed for IoT solutions aimed to benefit the University community [9–11].

In this rich context, we here present our testbed, designed, implemented, and deployed to assist administrative staff, ICT staff, faculty members, and, lastly, students, in increasing the sustainability and safety of the Cesena (Smart) Campus, one of the campuses of the University of Bologna. In particular, as a case study, we exploited a responsive web-based dashboard to analyze data by interacting directly with a visual representation of them. Such data are related to the University timetabling and are the result of the aggregation/integration of different data sources, including University Open Data and data gathered by our IoT infrastructure. In fact, the IoT infrastructure enables the collection of real-time data about i) premises (i.e., classrooms and labs) occupancy thanks to a camera-based people counting service [12]; and ii) environmental conditions (e.g, air quality, temperature, humidity) through the Canarin II sensors stations [13]. We deployed such IoT infrastructure in 20 classrooms and labs. To present the potentialities of our approach, two cases are presented: one related to sustainability and energy saving, and the other related to safety and the new COVID-19 restrictions and regulations.

The remaining of the paper is organized as follows. The next Section describes studies that investigate i) Data Visualization strategies in smart campus scenarios, ii) IoT infrastructures to measure the number of people in a Campus premise, and iii) IoT infrastructures and Smart Environment strategies to monitor occupancy and physical distances in indoor environments in the COVID-19 era. Inspired by these studies, we designed and implemented our system, as presented in Section 3. Then, we detail the dashboard we designed and implemented (Section 4) and, to explore the potentialities of such system, we present two cases study, focusing on sustainability and safety (Section 5).

## Related work

This Section briefly describes the most interesting research works related to the following topics: i) Data Visualization strategies in smart campus scenarios, ii) IoT infrastructures to measure the number of people in a Campus premise, and iii) IoT infrastructures and Smart Environments strategies to monitor occupancy and physical distances in indoor environments in the COVID-19 era.

### Data visualization solutions in the smart campus context

Considering Data Visualization as a tool to enable a better understanding of data in Smart Campus scenarios, dashboards can be an effective graphical user interface tool to provide at-a-glance views of the gathered data to users. In [14], authors created Campus Energy Education Dashboard (CEED), a dashboard to visualize the energy consumed inside a university campus to improve energy efficiency and increase the knowledge of its occupants. CEED used a digital map of the campus to provide information about the building that consumes more energy and a bar chart to display the same information. Exploiting both real-time and historical data. The system targets two different groups of users, providing analytic features for stakeholders and engagement features to students and staff. Another example is Ubidots, a dashboard where data from smartphones are collected and then visualized to monitor the mobility inside the campus [15]. In [16], Longre et al. proposed a model for the design of dashboards that display sensors data visualization in the context of a smart campus. In particular, they claim that the data displayed and the visualizations should take into consideration the users’ roles. Sensor data could be environmental-related. For example, Hentschel et al. used a simple web dashboard to display data related to temperature and light intensity inside an office at the University of Glasgow [17]. Instead, USC AiR is a mobile application to display data about the air quality inside a campus. This application also exploits augmented reality to make users more engaged with those data and to inspire them to contribute to the reduction of air pollution [18]. Similarly, Bujary et al. [19] resorted to augmented reality to lure users into using a pedestrian navigation application whose goal is also to gather quality environmental data in the premises of the University of Padua. The application is coupled with a web service providing a heatmap of collected historical values and their evolution in time. It was then expanded to be used even on smartwatches and with an algorithm able to generate best routes considering also light and brightness beside the classic shortest path [20]. AlmaMap is another example of an application that displays environmental data, such as temperature, humidity, pressure, and particulate matter, gather from sensors inside a university campus, whose location is visible through the visualization of the campus map [9]. Tarabieh et al. used a map-based visualization, together with a bar chart, pie chart, and dial gauge, to provide data about the energy consumption in a university campus and to produce a behavioral change in the campus community [21].

Focusing on the issue to detect and monitor classroom occupancy, Sutjarittham et al. used data visualization techniques, like heatmap and line chart, to display attendance patterns during classes and the actual occupancy in the classrooms [22]. Inspired by this study, we pushed this approach further, providing a data visualization dashboard that not only visualizes the classrooms/labs actual occupancy but also allows the user to easily perform visual comparisons to find the optimal course timetabling for real-time or medium- and long-term actions.

### IoT infrastructures for classroom occupancy monitoring

This SubSection focuses on some interesting approaches used to count people in indoor environments. In literature, there are many works devoted to indoor track and count persons, based on different technologies and architectural solutions. Some of those works are based on the use of passive infrared sensors (PIR) [23], which are electronic sensors that can measure infrared light radiating from some objects in their field of view. Some projects take advantage by using multiple PIR sensors [24], thus they can calculate the number of persons who are in an indoor environment by computing the number of people passing through doorways to access that place. A different approach is based on the Radio Frequency Identification (RFID) technology [25]. Basically, it consists of three components: (i) the readers, (ii) the tags, and (iii) the middleware software. Several solutions have been adopted and developed by applying this approach, exploiting probabilistic estimators that achieve the required accuracy and confidence level [26]. Other methods are based on the Wi-Fi probe-request-frame, which takes advantage of the fact that smartphones and mobile devices have been designed to periodically transmit such frames with the aim of identifying when a known access-point is positioned within a specific distance and by capitalizing such a kind of Wi-Fi behavior [27]. Hence, monitoring and counting these Wi-Fi frames can provide support in people counting and crowd monitoring [28]. A different process, based on the use of a single carbon dioxide sensor (CO_2_), has also been investigated [29]. The idea behind this approach is that an indoor environment is affected by human activities and that influence can be measured by various sensors, so as to infer the density of the crowd, as described in [30], or with the use of hybrid techniques, by combining different sensors such as video camera and CO_2_ sensors, as proposed in [31].

An additional approach we have analyzed is based on taking pictures by cameras: its main target is the design and the development of algorithms aiming at automatically counting people. A technique that could be adopted is based on the recognition of video segments stream, where the counting process can be divided into two main steps: (i) detecting moving blob on the basis of algorithms for motion detection (background subtraction and/or a segmentation strategy, i.e., K-means); (ii) monitoring the detected blobs, with the aim of identifying the direction of the monitor or to compute the number of people that are present in case of a single frame shot from a camera. In order to accomplish this task, two main categories of methods can be adopted: (i) the Line of Interest (LOI) counting methods can evaluate the number of people crossing a virtual Line of Interest within the monitored scene [32]; (ii) the Region of Interest (ROI) counting methods can estimate crowd, evaluating the number of people who are present within a specific Region of Interest in the monitored scene [33].

### IoT infrastructures and smart environments for occupancy monitoring in the COVID-19 era

This SubSection briefly introduces some of the main interesting and recent works related to the use of IoT and Smart Environments strategies with the aim of monitoring occupancy of indoor spaces and detecting physical distances among persons. The advent and the evolution of the COVID-19 pandemic have pushed research and development actions in this direction, with this specific goal. One of the technologies that have been mostly investigated has been passive WiFi sensing, so as to detect students’ and staff’s occupancy and mobility within a Campus and then analyzing how COVID-19 has impacted the related Control Policies [34]. In particular, Zakaria et al. have analyzed data collected from three different college campuses (two in Singapore and one in the Northeastern United States). Such a technology has been effective in monitoring the number of people in a building, on average, in a specific hour, and in detecting the number of places visited by a single person, on average, in a specific hour.

The context of university campuses is one of the most used case studies in such a situation. Longo et al. [35] present a prototype of a smart object (named Smart Gate), with the aim of monitoring people flow and of keeping track of the occupancy levels. Such a prototype is equipped with passive sensors (Time-of-Flight long-ranging sensors) and cameras, collecting data on a Raspberry Pi and transmitting them to a server for advanced analysis.

A monitoring system based on PIR (passive infrared radiation) sensors is proposed in [36], where the authors investigated the applicability of large data acquired from IoT tools for enhanced space use management to achieve operational efficiency at universities. The proposed study aims to identify methods for measuring and quantifying space use, that could significantly change during special circumstances such as the COVID-19 pandemic. Also in this study, the data collected from the IoT infrastructures are turned into information to support the decision-making on the management of campus, taking into account the Umea University (Sweden) as a case study.

In [37], Fernández-Caramés et al. proposed the use of Distributed Ledger Technologies (DLTs) including blockchain [38], together with Artificial intelligence algorithms with the aim of collecting data about people’s occupancy in an indoor environment, in a way that preserves privacy, and that is the most accurate, transparent and trustworthy as possible. The work proposed by these authors aims to estimate the real-time people occupancy level in public spaces such as buildings, classrooms, businesses, or moving transportation vehicles. In contrast to smartphone-based contract tracing applications (requiring an active involvement from the users, for instance by starting a mobile app or enable Bluetooth communications), such a system is based on autonomous wireless devices, without the need of an active user action after powering them on.

A project that exploits such technologies in an outdoor context is presented in [39], where the authors studied outdoor pedestrian activity in New York City. To create a holistic view of the city, the authors collected data in three different scales: (a) The City scale (data have been gathered through video streams gathered from public traffic cameras), (b) the Public scale (data have been gathered through video footage taken from instrumented vehicles driving through the city), and (c) the Individual scale (data have been gathered through mobile phone location data volunteered by local citizens). These multi-scale data will be used to form a map of urban mobility and space occupancy under social distancing policy.

## The system at a glance

**Fig. 1:**
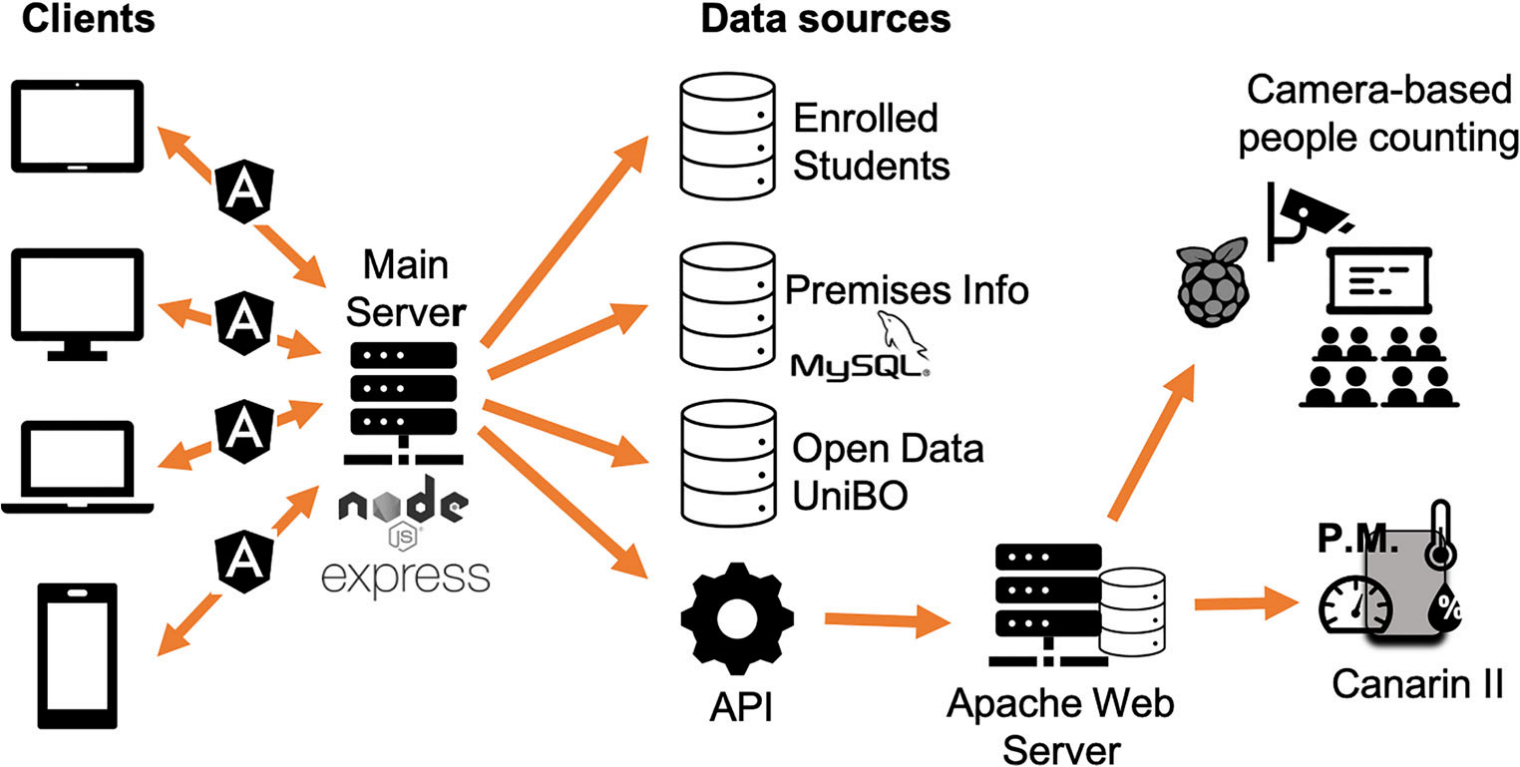
The system architecture

In this Section, we briefly present the technological aspects of the two core components of our approach: i) the IoT infrastructure to gather real-time classrooms/labs occupancy and environmental conditions, and ii) the Data Visualization web application. An overview of the overall architecture is depicted in Fig. 1. For more details about the design process, see [40].

### The IoT infrastructure

Our IoT infrastructure is composed of i) an edge-based system that exploits cameras for people counting, and ii) environmental conditions sensors stations to sense air contaminants, gathering formaldehyde, PM_1.0_, PM2.5 and PM10 values, temperature, relative humidity, air pressure.

Focusing on the camera-based system for people counting, the main constituent of our architecture is the client-side since almost the whole computation occurs there. In particular, the architecture is composed of three layers. (1) The data acquisition layer is devoted to the data acquisition, exploiting Intel RealSense D415 Depth cameras. The cameras are plugged-in (via USB) to a Raspberry Pi 4 model B and we acquired 1280 x 720 pixel frame rate images every five minutes (this interval was set to better support the storing operations). (2) The prediction layer retrieves data from the cameras, on the client-side, and exploits a custom model based on YOLOv3 with the aim of predicting the number of people in a precise moment, dividing the image into regions and predicting bounding boxes and probabilities for each region. In doing that, we exploit a transfer learning methodology. Once the prediction is done, the number of people predicted and the timestamp are stored in a file with csv extension. (3) The API layer, which, over HTTPS, responds the needed information to the server requests. This three-layer architecture based on a *fat client-thin server* has four main advantages: higher scalability, working semi-offline, higher availability, and privacy compliant. Moreover, our edge-based transfer learning model allows obtaining an average accuracy equal to or greater than 91% (depending on the room layout).

In our IoT infrastructure we also integrated the Canarin II sensors stations [13]. Such stations allow to monitor different environmental conditions, including formaldehyde, PM1.0, PM2.5 and PM10 values, temperature, relative humidity, and air pressure.

In our testbed, we deployed our infrastructure in 20 premises of the Cesena Campus, including 4 laboratories and 16 classrooms.

### The web application

The Data Visualization web application we designed and developed is based on a client-server architecture, as shown in Fig. 1. The client-side was implemented with the Angular framework^1^ and with the standard web-based languages, like HTML5, CSS3, and TypeScript.^2^ Concerning the data visualization elements, we used some Javascript libraries like Google Charts,^3^ Chart.js,^4^ and D3.js^5^ to make the charts interactive. Using these technologies, we were able to develop a responsive web-based application, enjoyable both from smartphones and tablets and larger screens (i.e., laptop).

Regarding the server-side, we used Node.js^6^ and the framework Express^7^. The server communicates with different data sources, such as i) open data provided directly by the University of Bologna, to gain information about the courses and the class schedule inside the Cesena campus; ii) a MySQL database containing information (such as name, location, capacity) about classrooms and laboratories inside the campus; and iii) a database with information about the number of enrolled students for each teaching and courses of study. Moreover, our server exploits some APIs to obtain data about the actual presence of people inside a classroom or laboratory (gained by the camera-based people counting infrastructure) and the data about temperature, humidity, air pressure and particulate matter from the Canarin II sensor stations [13]. This architecture allows us to easily extend it, including new data sources and sensors (through their API).

## The web-based dashboard

The responsive web-based application has been designed as a dashboard that can be accessed both from smartphones, to have quick consultations, for example, to find empty classrooms or laboratories at a specific time, and from devices with larger screens to let the users perform more complex analysis. The application aims to improve the management of spaces and class schedules by means of five main functions/components that allow the user to visualize intuitive charts and perform analysis. Each page has a left-side vertical menu (where it is possible to select the specific component out of five) and a search input box on the top-right side. In the following, all the implemented components are presented in detail.

### Overview

**Fig. 2:**
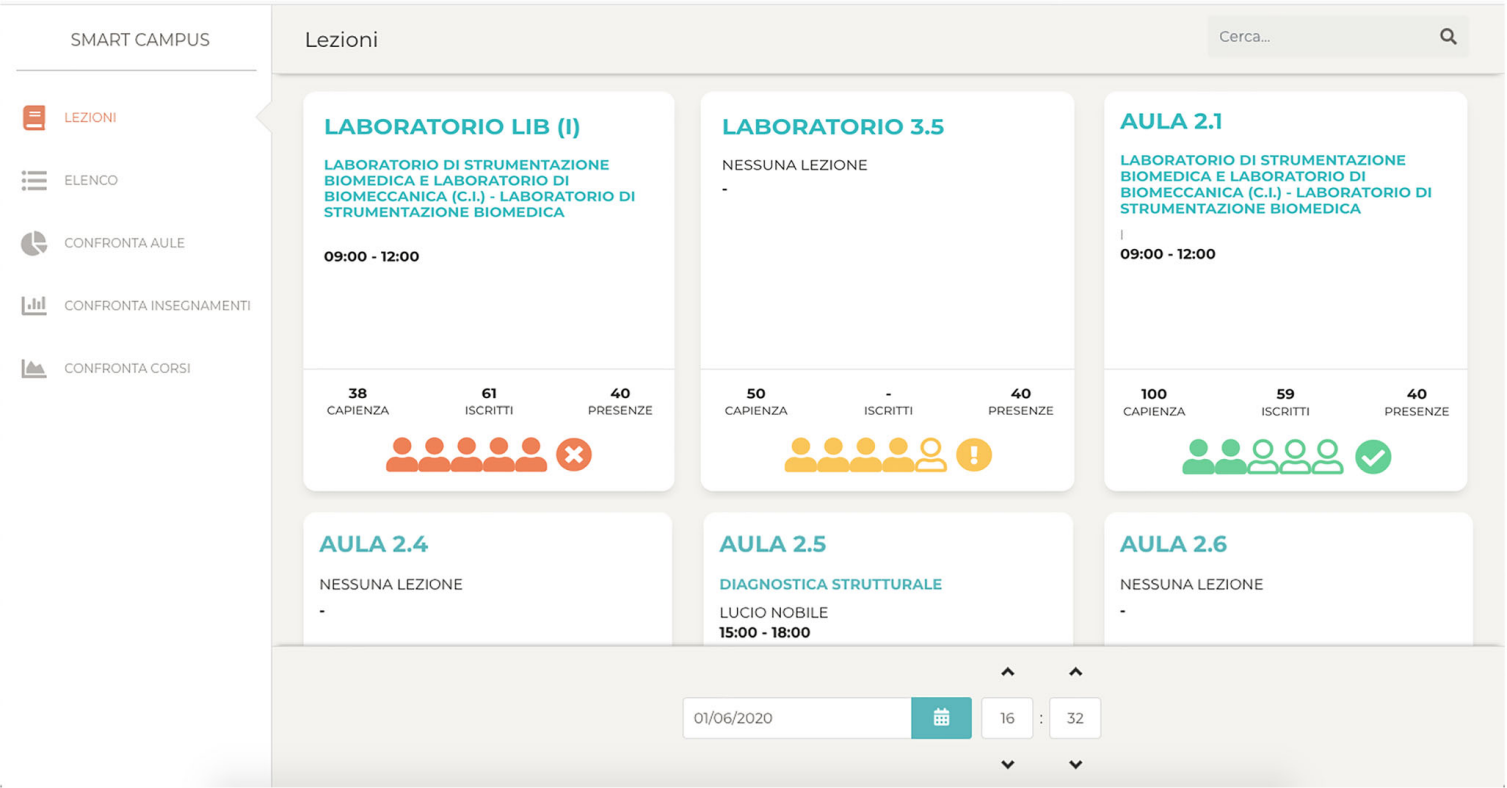
A screenshot of the system presenting the Overview component

The homepage of our web application opens the overview component (Fig. 2). This component displays a series of cards (e.g., three cards in Fig. 2), each card shows information about a classroom or lab, including details such as the room capacity and the room occupancy (at the specified timestamp). If a lesson is taking place, the card also shows the name of the course, the professor’s name, and the number of students enrolled. At the bottom of every card, there is a pictorial chart that highlights the occupation status of that room. The number of filled icons (out of five) represents the number of students inside the room in relation to its capacity. Adding more details, one filled icon corresponds to a room actual occupancy lower than 1/4 (25%) of the room capacity, two filled icons correspond to an occupancy lower than 2/4 (50%), and so on; the fifth icon is used only to warn that the room actual occupancy is equal or greater than the room capacity. Moreover, we take advantage of colors (green, yellow, and red) to convey messages and easily draw attention to anomalies. Accordingly, if the classroom or laboratory is too crowded (i.e., actual occupancy equal or greater than the facility capacity), all the five icons are filled in red, with an error icon beside it. Instead, in a normal situation, i.e., the actual occupancy is between 25% and 75%, the chart is green (with two or three filled icons), and, finally, if the condition has to be monitored by operators, i.e., lower than 25% (one filled icon) or greater than 75% (four filled icons), the chart is yellow with an alert icon beside it.

### List of classrooms, laboratories, courses, and teachings

The second component of our web application consists of four different lists, including all the classrooms, laboratories, courses, and degrees inside the Cesena campus, each list visualized in a card. The first two cards show the lists of the classrooms (in one card) and the laboratories (in another one) of the campus. Selecting one of them, the user can see the scheduled lessons through a simple list view or a timetable. For all the lessons already held in the selected classroom or laboratory, in the list view, it is also present the pictorial chart described in the overview component, while, in the timetable, the same information is conveyed through the background color of the cell representing the lesson duration over time. Moreover, we wanted to provide the users with an analysis tool to monitor the number of lessons, hours of teaching, and the percentage of usage of a given room (class or lab) through an easily understandable area chart. Lastly, we provide to the user the location of the room, using the campus map in SVG format. The third card in this component concerns the courses. For each of them, it is possible to see the general information, like the professor’s name and the degree program to which it belongs. The scheduled lessons are available both in a text list and area chart visualization. The two visualizations use different visual techniques to display the same information: the attendance trend, the number of enrolled students, and the capacity of the classrooms or laboratories where the lessons are held. The attendance rate is visible through the pictorial graph, in the list view, and through the color of the relative point in the area chart. To provide an additional tool for analysis, a stacked bar graph and a pie chart were included, presenting in green the percentage of spaces booked and actually used, in grey the percentage of lessons not yet held, and in red the percentage of spaces booked and not correctly used. The fourth and last card of this component regards the degree program, both bachelor’s and master’s degrees, in the Cesena campus. For each degree program, we choose to display a list view of all of the courses, the relative professor, and a statistics page to visualize the correct usage of booked spaces. Also in this case, we used a stacked bar chart and a pie chart with the same colors and the same meaning as the graphs described in the paragraphs above.

### Classrooms and laboratories usage comparison

The third component of the web application concerns the comparison between two or more classrooms and laboratories in terms of correct usage of space. As mentioned before, for each room (classroom and lab), we monitored the number of lessons and hours and the percentage of occupation. These elements provide a way to compare the effective usage of two or more different spaces. For each of the parameters considered, we used two different data visualization techniques (a line graph and a pie chart) to convey the same information so that the users can interact with the one that more reflects their demands. In particular, the line chart displays the number of lessons, hours, and the percentage of use in relation to the months of the year. Instead, the pie chart displays the same information but in relation to the total number of lessons, hours, and percentage of use.

### Courses comparison

**Fig. 3:**
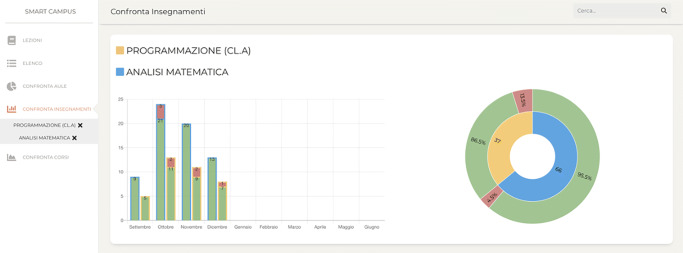
A screenshot of the system representing the Course comparison component

The comparison between two or more teachings/courses represents the fourth component of the application and it is presented in Fig. 3. To compare the different teachings, we used a stacked bar chart, and a sunburst graph to display the differences in terms of percentage of correct spaces usage and number of lessons. The stacked bar chart uses the border color to identify the course it refers to (in Fig. 3, yellow and blue), the green and red color of the bar to display the correct or incorrect usage of spaces, and the gray color for the lessons not yet held in relation to the months of the year. Concerning the sunburst graph, the first ring is dived according to the number of lessons for each teaching, while the second one represents the percentage of correct and incorrect spaces usage for each teaching.

### Degree programs comparison

Finally, the last component of our web-based application concerns the comparison of two or more degree programs. Also, in this case, we use the number of lessons and the percentage of correct and incorrect usage of spaces as parameters to compare the degree programs. We implemented a stacked bar chart and a sunburst graph to visualize the number of lessons and the usage of booked space . The meaning of the graphs and the use of border and bar colors is the same as in the course comparison component.

## Case studies

To present the potentialities of our system in supporting premises managements, we here present two case studies. The first focuses on using the data visualization system to verify the real-time occupancy considering the actual COVID restrictions and regulations; the second is related to a medium/long-term monitoring of the classrooms/labs occupancy to increase the sustainability of the campus in terms of optimization of the energy consumption.

### Case study 1: real-time safety monitoring

The COVID-19 pandemic changed the way we experience any space and place, put into effect social distancing measures. Considering University campuses, not always lessons can be taught in presence, but, if and when possible, in-person classes have to be taught in accordance with regulatory requirements and safety protocols. Different strategies have been adopted to ensure compliance with regulatory requirements and safety protocols. For example, the University of Bologna, to manage the capacity of each classroom/lab in the best possible way, developed a service that allows students to schedule the classes they plan to attend in two-week periods, specifying the attendance mode. Unfortunately, this strategy not always works as expected. For this reason, IoT can be exploited, along with similar solutions, to guarantee safety during in-person classes.

Exploiting our IoT infrastructure, and, in particular, the camera-based people counting system, we are able to monitor, in real-time, the number of students on a University premise. Thanks to our Data Visualization dashboard, we can obtain the actual classrooms/labs capacity (considering the current regulation), and visualize the room occupancy accordingly. This information is visible on the system home page, in the overview component. In fact, the card allows to immediately check the room occupancy in relation to the room capacity to ensure social distance and safety. As an example, going back to Fig. 2, used to presented the overview components, it is possible to infer that: in lab LIB (on the left), the actual occupancy is greater than the actual capacity (red color and five filled icons), while in lab 3.5 (center) the actual occupancy is close to the maximum (yellow color and four our of five filled icon), and, finally, in lab 2.1 (on the right), the number of students in the room doesn’t present any anomaly (green color and two out of five filled icons). When the pictorial chart becomes red (i.e., the room’s actual occupancy is greater than the room’s actual capacity), a notification is sent to the people responsible for the health and safety inside the campus.

**Fig. 4:**
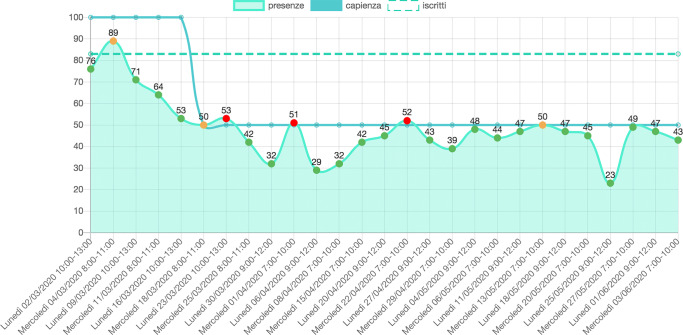
An area char representing the classroom occupancy values monitored during the COVID-19 pandemic, for a specific course

Moreover, by integrating University official data sources, the system can automatically update its data and visualize the information accordingly. As an example, in Fig. 4 it is possible to see the impact of the decrease of the allowed room capacity value due to COVID-19 regulations regarding the social distance (the allowed room capacity became 50, while was 100 by design).

### Case study 2: medium/long term sustainability

As mentioned, defining a school/university timetabling is a complex task to solve [41]. With our approach, we are not interested in automatically solving such a problem, but mostly to exploit data visualization and IoT strategies to facilitate University staff in taking the best decisions, considering also the campus sustainability. In fact, it has been proved that an efficient timetable could also reduce energy consumption and therefore increase the sustainability of the campus [42].

**Fig. 5:**
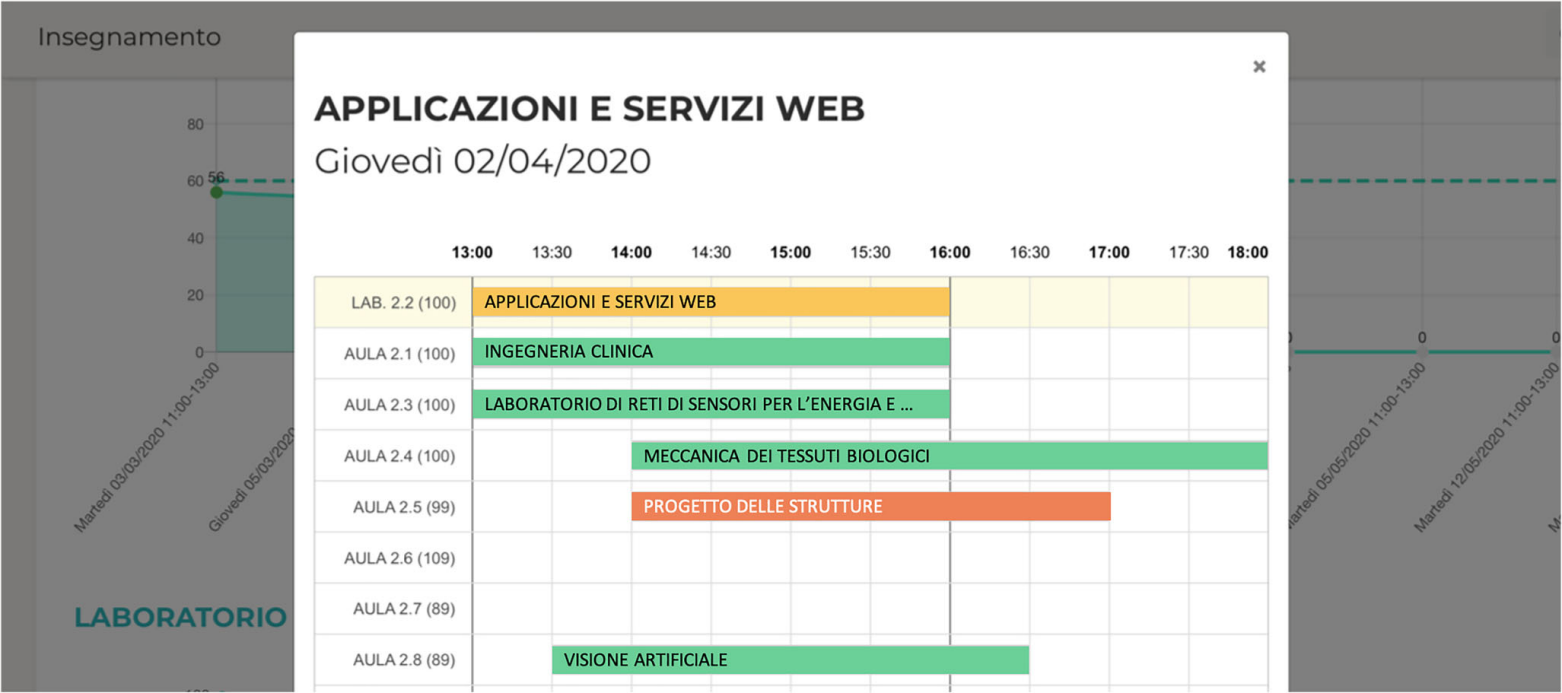
Timetable to compare classrooms (or labs) availability and perform an exchange to improve sustainability

Our visual application has been designed to facilitate the discovery of not optimal classrooms/labs and course assignment, on the basis of the real-time data collected by our IoT infrastructure. In fact, as presented also in Fig. 4, selecting one course, it is possible to analyze in detail the actual occupancy of the room during the course weeks, in relation to the room capacity. Moreover, by selecting one specific value in the chart, it is possible to see a modal presenting the timetable of the other courses and see the classrooms (or labs) availability during that time slot (as shown in Fig. 5). Considering, for example, that for the last three weeks the number of students attending a course is half than expected, it is possible to “release” the assigned room and look for a smaller room that can host the students. This action can have an immediate impact on energy saving because a large space causes a higher level of heating and cooling loads if compared with a smaller one.

Moreover, through the course comparison component (that we previously presented exploiting Fig. 3), it is possible to compare two courses, focusing on two values: the number of lessons that have achieved a satisfactory occupancy value in relation to the room capacity, and the number of lessons that prompted an anomaly. These visualizations could help the administrative staff to change the classroom for those lessons that have an attendance rate much lower or greater than the capacity of the room, leading to an improvement in space management on our campus.

## Conclusion and future work

In this paper, we present an integrated approach that combines IoT infrastructures (e.g., a camera-based people counting service and sensors for environmental conditions monitoring) and Data Visualization strategies to i) ensure safety, and ii) improve the sustainability of a University campus. As a testbed, we deployed our approach in the Cesena (smart) campus, one of the campuses of the University of Bologna. It is worth noting that the system was initially designed with the main goal of facilitating University staff to monitor premises occupancy and improve the course timetabling accordingly, increasing the Campus sustainability. Nonetheless, with the starting of the COVID-19 pandemic, it shows its potential to monitor safety aspects introduced with the actual regulations and constraints. As future work, we plan to integrate a machine learning module in our web application that uses the data gathered during the first year of deployment to suggest timetabling recommendations to improve Campus sustainability and safety.

## Footnotes

1 https://angular.io/

2 https://www.typescriptlang.org/

3 https://developers.google.com/chart

4 https://www.chartjs.org/

5 https://d3js.org/

6 https://nodejs.org/

7 https://expressjs.com/

## References

[1] Data Visualization. https://www.gartner.com/en/marketing/glossary/data-visualization. [Online; accessed 01-10-2020]

[2] Prandi C, Mirri S, Ferretti S, Salomoni P (2017) On the need of trustworthy sensing and crowdsourcing for urban accessibility in smart city. ACM Transactions on Internet Technology (TOIT) 18(1):1–21

[3] Palazzi CE, Bujari A (2016) Fostering accessible urban mobility through smart mobile applications. In: 2016 13th IEEE annual consumer communications & networking conference (CCNC), pp 1141–1145. IEEE

[4] Roccetti M, Marfia G, Palazzi CE (2011) Entertainment beyond divertissment: using computer games for city road accessibility. Computers in Entertainment (CIE) 9(2):1–9

[5] Prandi C, Ceccarini C, Nisi V, Salomoni P (2020) Designing interactive infographics to stimulate environmental awareness: an exploration with a university community. Mult Tools App, pp 1–18

[6] Abdelalim A, O’Brien W, Shi Z (2017) Data visualization and analysis of energy flow on a multi-zone building scale. Autom Constr 84:258–273. 10.1016/j.autcon.2017.09.012. http://www.sciencedirect.com/science/article/pii/S0926580516302813

[7] Kuutti J, Saarikko P, Sepponen RE (2014) Real time building zone occupancy detection and activity visualization utilizing a visitor counting sensor network. In: 2014 11th International conference on remote engineering and virtual instrumentation (REV), pp 219–224

[8] Muhamad W, Kurniawan NB, Yazid S, et al. (2017) Smart campus features, technologies, and applications: A systematic literature review. In: 2017 International conference on information technology systems and innovation (ICITSI), pp 384–391. IEEE

[9] Prandi C, Monti L, Ceccarini C, Salomoni P (2020) Smart campus: Fostering the community awareness through an intelligent environment. Mob Netw Appl 25(3):945–952. 10.1007/s11036-019-01238-2

[10] Nati M, Gluhak A, Abangar H, Headley W (2013) Smartcampus: A user-centric testbed for internet of things experimentation. In: 2013 16th International symposium on wireless personal multimedia communications (WPMC), pp 1–6. IEEE

[11] Liu X (2017) A study on smart campus model in the era of big data. In: Proceedings of the 2016 2nd International conference on economics, management engineering and education technology (ICEMEET 2016), pp 919–922. Atlantis Press. 10.2991/icemeet-16.2017.191

[12] Monti L, Mirri S, Prandi C, Salomoni P (2019) Smart sensing supporting energy-efficient buildings: On comparing prototypes for people counting. In: Proceedings of the 5th EAI international conference on smart objects and technologies for social good, pp 171–176

[13] Monti L, Prandi C, Mirri S (2018) Iot and data visualization to enhance hyperlocal data in a smart campus context. In: Proceedings of the 4th EAI international conference on smart objects and technologies for social good, pp 1–6

[14] Salmon K, Morejohn J, Sanguinetti A, Pritoni M (2016) The iterative design of a university energy dashboard. In: 2016 ACEEE summer studies on energy efficiency in buildings

[15] Alonso J, Bayona C, Rojas O, Tern M, Aranda J, Carrillo H, Parra C (2018) Iot solution for data sensing in a smart campus using smartphone sensors. In: 2018 IEEE Colombian conference on communications and computing (COLCOM), pp 1–6

[16] Logre I, Mosser S, Collet P, Riveill M (2014) Sensor data visualisation: A composition-based approach to support domain variability. In: Cabot J, Rubin J (eds) Modelling foundations and applications, pp 101–116. Springer International Publishing, Cham

[17] Hentschel K, Jacob D, Singer J, Chalmers M (2016) Supersensors: Raspberry pi devices for smart campus infrastructure. In: 2016 IEEE 4th International conference on future internet of things and cloud (FiCloud), pp 58–62

[18] Ramachandran GS, Bogosian B, Vasudeva K, Sriramaraju SI, Patel J, Amidwar S, Malladi L, Shylaja RD, Kumar NRB, Krishnamachari B (2019) An immersive visualization of micro-climatic data using usc air (demo). In: Proceedings of the 17th Annual International Conference on Mobile Systems, Applications, and Services, MobiSys ’19, pp 675–676. Association for Computing Machinery, New York, NY, USA. 10.1145/3307334.3328577

[19] Bujari A, Ciman M, Gaggi O, Marfia G, Palazzi CE (2015) Paths: Enhancing geographical maps with environmental sensed data. In: Proceedings of the 2015 workshop on pervasive wireless healthcare, pp 13–16

[20] Bujari A, Gaggi O, Palazzi CE (2020) A mobile sensing and visualization platform for environmental data. Pervasive and Mobile Computing 66:101204

[21] Tarabieh KA, Elnabarawy IO, Mashaly IA, Rashed YM (2015) The power of data visualization: A prototype energy performance map for a university campus. In: Sustainable Human–Building Ecosystems, pp 194–203. https://ascelibrary.org/doi/abs/10.1061/9780784479681.021

[22] Sutjarittham T, Gharakheili HH, Kanhere SS, Sivaraman V (2018) Realizing a smart university campus: Vision, architecture, and implementation. In: 2018 IEEE International conference on advanced networks and telecommunications systems (ANTS), pp 1–6

[23] Yun J, Lee S-S (2014) Human movement detection and identification using pyroelectric infrared sensors. Sensors 14(5):8057–8081 24803195 10.3390/s140508057 PMC4063065

[24] Raykov YP, Ozer E, Dasika G, Boukouvalas A, Little MA (2016) Predicting room occupancy with a single passive infrared (pir) sensor through behavior extraction. In: Proceedings of the 2016 ACM international joint conference on pervasive and ubiquitous computing, pp 1016–1027

[25] Angeles R (2005) Rfid technologies: supply-chain applications and implementation issues. Inf Syst Manag 22(1):51–65

[26] Kodialam M, Nandagopal T (2006) Fast and reliable estimation schemes in rfid systems. In: Proceedings of the 12th annual international conference on mobile computing and networking, pp 322–333

[27] Yaik OB, Wai KZ, Tan Ian KT, Sheng OB (2016) Measuring the accuracy of crowd counting using wi-fi probe-request-frame counting technique. (JTEC) 8(2):79–81

[28] Li K, Yuen C, Kanhere SS, Hu K, Zhang W, Jiang F, Liu X (2018) Understanding crowd density with a smartphone sensing system. In: 2018 IEEE 4th World forum on internet of things (WF-IoT), pp 517–522. IEEE

[29] Longo S, Cheng B (2015) Privacy preserving crowd estimation for safer cities. In: Proceedings of the 2015 ACM international joint conference on pervasive and ubiquitous computing and proceedings of the 2015 ACM international symposium on wearable computers - UbiComp ‘15. 10.1145/2800835.2801631. ACM Press

[30] Li T, Fong S (2018) Counting passengers in public buses by sensing carbon dioxide concentration: System design and implementation. In: Proceedings of the 2018 2nd international conference on big data and internet of things, pp 218–221. ACM

[31] Wang F, Feng Q, Chen Z, Zhao Q, Cheng Z, Zou J, Zhang Y, Mai J, Li Y, Reeve H (2017) Predictive control of indoor environment using occupant number detected by video data and CO 2 concentration. Energy and Buildings 145:155–162. 10.1016/j.enbuild.2017.04.014

[32] Luna CA, Losada-Gutierrez C, Fuentes-Jimenez D, Fernandez-Rincon A, Mazo M, Macias-Guarasa J (2017) Robust people detection using depth information from an overhead time-of-flight camera. Expert Syst Appl 71:240–256

[33] Fernandez-Rincon A, Fuentes-Jimenez D, Losada-Gutierrez C, Romera MM, Luna CA, Guarasa JM, Mazo M (2017) Robust people detection and tracking from an overhead time-of-flight camera.. In: VISIGRAPP (4: VISAPP), pp 556–564

[34] Zakaria C, Trivedi A, Chee M, Shenoy P, Balan R (2020) Analyzing the impact of covid-19 control policies on campus occupancy and mobility via passive wifi sensing. arXiv:2005.12050

[35] Longo E, Redondi Alessandro EC, Bianchini M, Bolzan P, Maffei S (2020) Smart gate: a modular system for occupancy and environmental monitoring of spaces. In: 2020 5th International Conference on Smart and Sustainable Technologies (SpliTech), pp 1–6. IEEE

[36] Azizi S, Nair G, Rabiee R, Olofsson T (2020) Application of internet of things in academic buildings for space use efficiency using occupancy and booking data. Build Environ 186:107355 33041459 10.1016/j.buildenv.2020.107355 PMC7531345

[37] Fernández-Caramés TM, Froiz-Míguez I, Fraga-Lamas P (2020) An iot and blockchain based system for monitoring and tracking real-time occupancy for covid-19 public safety. In: Engineering proceedings, vol 2, pp 67. Multidisciplinary digital publishing institute

[38] El Ioini N, Pahl C (2018) A review of distributed ledger technologies. In: OTM Confederated International Conferences” On the Move to Meaningful Internet Systems”, pp 277–288. Springer

[39] Ju W, Yavo-Ayalon S, Mandel I, Saldarini F, Friedman N, Sibi S, Zamfirescu-Pereira JD, Ortiz J (2020) Tracking urban mobility and occupancy under social distancing policy. Digital Government: Research and Practice 1(4):1–12

[40] Ceccarini C, Mirri S, Prandi C, Salomoni P (2020) A data visualization exploration to facilitate a sustainable usage of premises in a smart campus context. In: Proceedings of the 6th EAI international conference on smart objects and technologies for social good, GoodTechs ’20, pp 24–29. Association for Computing Machinery, New York, NY, USA. 10.1145/3411170.3411241

[41] Pandey J, Sharma AK (2016) Survey on university timetabling problem. In: 2016 3rd International conference on computing for sustainable global development (INDIACom), pp 160–164. IEEE

[42] Song K, Kim S, Park M, Lee H-S (2017) Energy efficiency-based course timetabling for university buildings. Energy 139:394–405

